# Effects of a Visual Distracter Task on the Gait of Elderly versus Young Persons

**DOI:** 10.1155/2011/651718

**Published:** 2011-06-16

**Authors:** Otmar Bock, Rainer Beurskens

**Affiliations:** Institute of Physiology and Anatomy, German Sport University, 50933 Cologne, Germany

## Abstract

Seniors show deficits of dual-task walking when the second task has high visual-processing requirements. Here, we evaluate whether similar deficits emerge when the second task is discrete rather than continuous, as is often the case in everyday life. Subjects walked in a hallway, while foot proprioception was either perturbed by vibration or unperturbed. At unpredictable intervals, they were prompted to turn their head and perform a mental-rotation task. We found that locomotion of young subjects was not affected by this distracter task with or without vibration. In contrast, seniors moved their legs after the distraction at a slower pace through smaller angles and with a higher spatiotemporal variability; the magnitude of these changes was vibration independent. We conclude that the visual distracter task degraded the gait of elderly subjects but completely spared young ones, that this effect is not due to degraded proprioception, and that it rather might reflect the known decline of executive functions in the elderly.

## 1. Introduction

The human gait pattern is affected by old age. For example, walking speed and stride length decrease, while lateral sway, foot velocity at ground contact, and stride time variability increase [1–5]. Some of these changes are compensatory in that they stabilize body posture, while others are dysfunctional and correlate with the risk of accidental falls [6, 7]. The observed deficits have been attributed in literature to a variety of causal factors, notably to cognitive decline; indeed, the critical role of cognition is supported by the fact that age-related gait changes are more pronounced in persons with cognitive impairment [8, 9] and that they are accentuated under dual-task conditions [10, 11].

We have recently compared single- and dual-task gait of young and elderly subjects with 14 different combinations of a walking and a nonwalking task and found age-related deficits of dual-task gait for some but not for other combinations. Specifically, we observed deficits whenever the non-walking tasks required continuous visual processing and no deficits without such a requirement, irrespective of the difficulty of the walking and the nonwalking task [12–15]. We attributed this finding to the well-known shrinkage of prefrontal gray matter in advanced age and the associated decline of executive functions [16, 17]. According to this view, walking relies on continuous visual processing to control heading and avoid obstacles as we navigate through a visually defined environment [18–20]; when a visual nonwalking task is added, two streams of visual information must be managed concurrently, which could exceed the capacity of an aging prefrontal cortex. Experimental evidence confirms that indeed, processing of visual information for postural control and obstacle avoidance interferes with the processing of visual information for a concurrent task [21–24] and that this interference is more pronounced in old age [22, 23] particularly in seniors with a history of falls [23]. It has also been shown that seniors' likelihood to fall is associated with deficits of executive functions [11, 25].

Our above findings were yielded in typical laboratory experiments, but they could have implications for everyday life. For example, persons walking outdoors may have to watch for traffic or may be distracted by advertisement boards and shop window displays. Likewise, persons walking at home may balance a cup of coffee in their hand or may be distracted by visitors, pets, and information displayed on their TV set. All these scenarios require that multiple streams of visual information are handled concurrently, which may overtax seniors' abilities and thus precipitate accidental falls.

The above generalization from laboratory experiments to real life might be flawed however dual-task experiments revealed age-related deficits for tasks that require continuous visual processing, while everyday events often need brief bouts of visual processing, and thus might be easier to integrate with the walking task. To find out, the present study evaluates whether brief and unpredictable distracters have more dramatic effects on the gait pattern of healthy elderly subjects if compared to healthy young subjects.

## 2. Methods

### 2.1. Subjects

Twelve young and twelve older subjects participated; their gender and anthropometric characteristics are summarized in Table 1. All subjects were free of gait or other orthopedic disorders; subjects who wore corrective eyeglasses upon arrival continued to wear them during the experiments. All participants lived independently in the community and had not participated in research on gait or cognition within the preceding six months. All signed an informed consent statement for this study, which was pre-approved by the authors' institutional Ethics Committee.

### 2.2. Walking and Mental-Rotation Tasks

The experiments were carried out in a 2.5 m wide hallway on the floor of which a straight path of 20 m length and 1.8 m width was marked by red-and-white tape. Eight 17′′-monitors were arranged at irregular intervals parallel to the wall, four to the left and four to the right; their center was 116.5 cm above ground. Subjects walked the path ten times back and forth at their preferred speed, thus covering a total distance of 400 m and passing 160 times by a monitor. On twelve of those passes, they heard the command “left” or “right,” referring to the location of the upcoming monitor; at the same time, a capital letter from the Latin alphabet was displayed for 2 s on that monitor. To exclude any difficulty in perceiving and recognizing the letters, they were of large size (12.5 cm height) and high contrast (black on white background). During successive trials, different asymmetrical letters (such as “G” or “K”) were presented mirror-reversed or non-reversed, at a rotation angle of ±60% or ±120° with respect to the vertical; the same, quasirandom sequence of letters was used for all subjects. This acoustic-and-visual stimulus was triggered by the subjects' first heelstrike at less than 1.3 m distance from the pertinent monitor.

The twelve monitor passes with stimulus presentation were selected among all 160 passes according to a quasirandom sequence, with the constraint that during each transit along the 20 m walking path, either no, one, or two stimuli were triggered; the same sequence was used for all participants. We instructed the subjects to respond to each letter by saying “yes” if it was mirror-reversed, and “no” if it was non reversed. It is known that in such a task, the letters are first mentally rotated into the upright before a judgment is made regarding the presence or absence of reversal [26]. Since the letters were presented during locomotion in our study, subjects invariably turned their head towards the monitor before making a decision. However, they did not stop walking and did not appreciably rotate their trunk on any trial.

To manipulate sensory feedback from the subjects' feet, four battery-operated vibrators were attached by elastic bands to the tendons of M. soleus and M. tibialis anterior of each leg. Subjects completed the above procedures once in condition NOVIB, with the vibrators turned off, and once in condition VIB, with the vibrators operating at a frequency of 80 Hz and an amplitude of about 1 mm. Such a stimulation of antagonistic muscles does *not* induce tonic vibration reflexes or movement illusions [27, 28], but it masks afferent inputs from distal limb segments, thus dramatically reducing the sense of position, touch and force, as well as H-reflex magnitude [29–31].

### 2.3. Data Registration and Analysis

Subjects' performance was registered with the MTx orientation tracking system (Xsens Technologies, NL). Four sensors were mounted with Velcro strips to the thigh and shank of each leg and a fifth one to the subjects' right temple. Sensor signals were sent by wireless transmission to a stationary computer, which determined in real time the orientation of each sensor in the sagittal plane with a sampling rate of 100 Hz and an accuracy of better than 1°. Individual step cycles were subsequently identified by a recursive-correlation algorithm, which detected the repetition of 390 ms data segments of similar shape [13, 14]. We then determined two gait measures for each step cycle of the lower right leg: *step duration* as the interval from step onset to end, and *leg rotation* as the difference between the maximum and minimum orientation angle of the lower right leg. Both measures were subsequently sorted with respect to the step which triggered stimulus presentation, starting with the fifth step before and ending with the eighth step after the trigger. We then calculated for each of these 13 steps the mean value and the coefficient of variation (CV) of each gait measure separately for each subject. The outcome—two means and two CVs—was submitted to separate three-way analyses of variance (ANOVAs), with grouping factor age and with repeated measures on the factors step and vibration.

We further determined, for each stimulus presentation, several measures of head movement. *Reaction time* was defined as the interval between stimulus appearance and movement onset, *duration* as the interval between movement onset and end, *speed* as peak movement velocity, and *head angle* as the maximum angle of head rotation. The means of each measure across stimulus presentations were submitted to two-way ANOVAs with the grouping factor age and with repeated measures on vibration. 

Subjects' verbal responses to the displayed letter were occasionally wrong. An observer tallied those errors, and we subsequently submitted the *error rates* to an ANOVA with the between factor age and the within factor vibration.

### 2.4. Cognitive Tasks

On a separate day, we assessed subjects' *psychomotor speed* as simple manual reaction time to visual stimuli presented at irregular intervals. We also quantified subjects' *visuoconstructive skill*, *visual planning skill*, and *visual memory* as scores on subtests of the German intelligence test IST2000R [32], and *executive functions* as reaction times in a modified Stroop task [14]. In the latter task, the words “gelb” (*yellow*) or “grün” (*green*) were presented in the center of a computer screen in yellow or green color. Subjects were asked to respond as quickly as possible to yellow stimuli by pressing a button with their right hand and to green stimuli by pressing a button with their left hand. This instruction was fostered by the continuous display of a yellow bar along the right and a green bar along the left edge of the screen. The color and meaning of words was congruent in one block of 55 trials but incongruent in another block of 55 trials. In the incongruent block, subjects had to respond according to the color when a word was presented against a black background and according to the meaning when a word was presented against a gray background. The reaction time difference between congruent and incongruent block was taken as a measure of the subjects' ability for inhibiting preferred responses and for rule switching.

The cognitive scores of young and older subjects were compared by *t*-tests. The relationship between gait pattern on the one side, and head movements, cognition and age on the other side, was explored by stepwise multiple linear regression analyses (MLR). The four gait measures served as dependent variables, and all head-movement and cognition measures as regressors. Age was converted to a regressor by setting older =1 and younger =0. The criterion for including and excluding a regressor in the stepwise analysis was set to *F* > 1.

## 3. Results 

**Figure 1:**
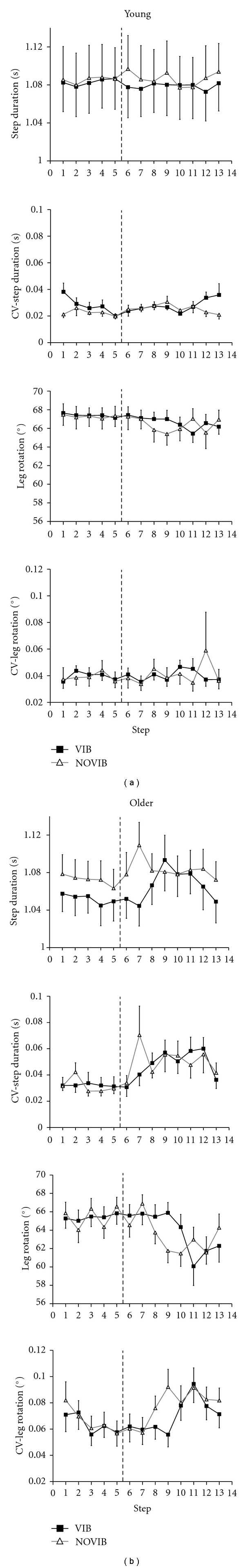
Gait parameters in young (a) and elderly subjects (b) during the last five steps before and the first eight steps after a distracter stimulus (dashed lines). Symbols represent across-subjects means and error bars the corresponding standard errors. Subjects walked without vibration (squares, black lines) as well as with vibration of the pronator and supinator muscle tendons of both feet (triangles, gray lines).

Figure 1 depicts the values of all four gait measures for the last five steps before, and the first eight steps after, stimulus onset. To facilitate comparisons, conditions NOVIB and VIB are plotted together while the two age groups are graphed in separate columns. Clearly from Figure 1, the gait of young subjects changed little after stimulus appearance whether or not the feet were vibrated. In contrast, the walking pattern of older subjects was distinctly modified by the stimulus: mean step duration decreased, while mean step angle and the spatiotemporal variability of steps increased. These changes were most pronounced during the 4th to 6th step after stimulus onset in condition NOVIB and about 2 steps earlier in condition VIB. The magnitude of changes was comparable in both conditions. Accordingly, three-way ANOVA for mean *step duration* yielded significant effects of step ∗ age (*F*(12,264) = 3.29; *P* > .05), step ∗ vibration (*F*(12,264) = 3.52; *P* < .001) and step ∗ vibration ∗ age (*F*(12,264) = 2.48; *P* < .01). For mean *leg rotation*, we found significant effects of step ∗ age (*F*(12,264) = 5.46; *P* < .001), step ∗ vibration (*F*(12,264) = 5.99; *P* < .001) and step (*F*(12,264) = 16.84; *P* < .001). For the CV of *step duration*, significance emerged with respect to step (*F*(12,264) = 4.77; *P* < .001), age (*F*(1,22) = 17.31; *P* < .001) and step ∗ age (*F*(12,264) = 3.52; *P* < .001), and for the CV of *leg rotation* also with respect to step (*F*(12,264) = 3.22; *P* < .001), age (*F*(1,22) = 12.52; *P* < .01) and step ∗ age (*F*(12,264) = 2.14; *P* < .05).

**Figure 2:**
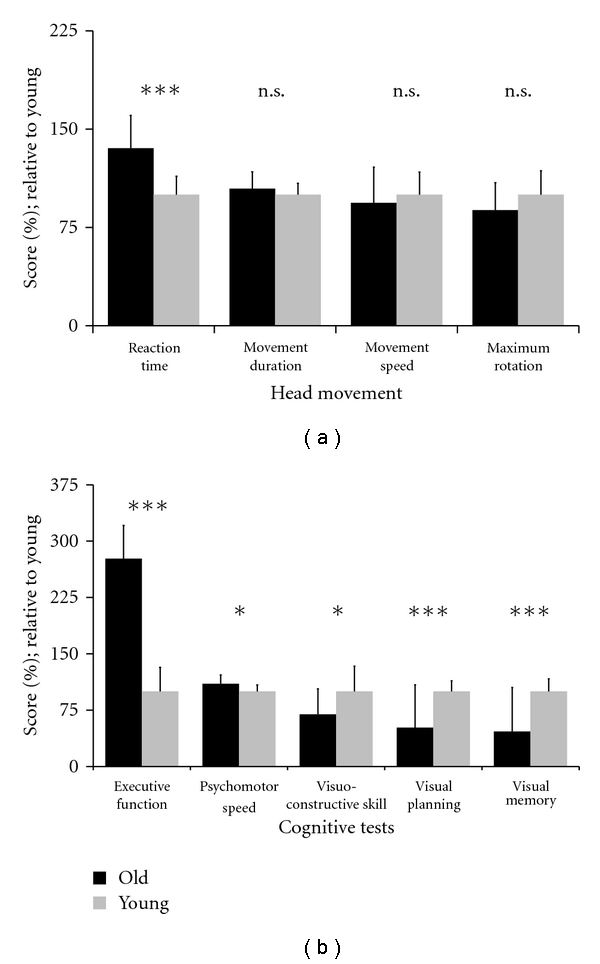
Measures of head movement (a) and of cognition (b). To facilitate comparisons, raw scores were transformed into percentages of young subjects' means. The means of older subjects are depicted by black blocks, and those of young subjects (i.e., 100%) by gray blocks. Error bars represent the standard errors ***indicates *P* < .001, *indicates *P* < .05, and n.s. indicates no significance. Note that “executive function” and “psychomotor speed” are time based parameters such that lower scores indicate better performance.

Figure 2(a) illustrates the head movement measures of young and older subjects. Two-way ANOVA of those data yielded only one significant effect that of age on *reaction time* (*F*(1,22) = 18.31; *P* < .001). Thus, young subjects initiated their head movements with a shorter delay than older ones; this age difference averaged 0.13 s across subjects and conditions. Two-way ANOVA of *error rate* yielded only a significant effect of Age (*F*(1,22) = 4.32; *P* < .05).  Figure 2(b) shows that young subjects outperformed older ones on all cognitive tasks, which we confirmed by *t*-tests (*executive function*: *t*(22) = 4.85; *P* < .001, *psychomotor speed*: *t*(22) = 2.30; *P* < .05, *visuoconstructive skill*: *t*(22) = −2.58; *P* < .05, *visual memory*: *t*(22) = −5.12; *P* < .001, *visual planning*: *t*(22) = −5.79; *P* < .001). 

To simplify further analyses, we replaced the original gait measures by the difference Δ*g* between the last step before stimulus onset (i.e., step 5) and that subsequent step which showed the largest impact of the stimulus (i.e., step 7 for the mean and CV of *step duration* in condition VIB, step 11 for the mean and CV of *leg rotation* in condition NOVIB, and step 9 otherwise; cf. Figure 1). ANOVA with the grouping factor age and repeated measures on Vibration yielded only significant effects of age on all Δ*g* (mean step duration: *F*(1,22) = 24.74; *P* < .001, CV of step duration: *F*(1,22) = 5.01; *P* < .05, mean leg rotation: *F*(1,22) = 17.59; *P* < .001, CV of leg rotation: *F*(1,22) = 9.70; *P* < .01). In other words, the presence or absence of foot vibration had no reliable effect on the magnitude of stimulus-induced gait changes Δ*g*. We, therefore, decided to average Δ*g* across VIB and NOVIB for our final set of analyses.

Table 2 shows that simple linear regression of Δ*g* on *age* was significant for all four Δ*g*, which confirms that the effects reported above for the ANOVA-factor Age can be replicated with the regressor *age*. Further, from Table 2, stepwise MLR eliminated the effects of *age *on two  Δ*g* measures, replacing them with cognitive measures of *executive function* (for both  Δ*g*) and *visual memory* (for one Δ*g*). The effects of *age* persisted for the other two Δ*g* measures but were supplemented by the effects of *executive function* (for one  Δ*g*) and *psychomotor speed* (for both  Δ*g*). Thus, the stimulus-induced gait changes of our elderly subjects are at least partly predictable from their cognitive performance, notably that captured by our modified Stroop task.

## 4. Discussion

The present study investigated the effects of unpredictable distractions on the gait pattern of young and elderly subjects walking at their preferred speed. We attempted to model the distractions after events in everyday life: an acoustic signal prompted the subjects to turn their head, to perform complex visuospatial processing, and finally to select an adequate response. A similar sequence occurs in real life, for example, when a person crossing the road hears an approaching car and turns the head, assesses the likelihood of a collision, and then decides to raise the arm such as to make the driver aware of her presence. Obviously, the particular acoustic signals, visual processing requirements, and potential responses are not the same for our distracter and for the outlined everyday event, but they differ between individual real-life events as well.

We observed no effects of distracters on the spatiotemporal gait characteristics of young subjects not even after degrading the sensory information from their feet. In contrast, we found considerable effects on the gait of elderly subjects: the legs moved at a slower pace through smaller angles, and the spatiotemporal variability increased. These changes did not emerge immediately after stimulus onset but rather were delayed: Figure 1 shows that they peaked 1-2 s, or 3-4 s, or even 5-6 s after the stimulus, depending on condition and gait measure. For comparison, the head rotated towards the monitor within about 2.3 s (sum of mean reaction and movement time in seniors: 2.32 s for NOVIB, 2.26 s for VIB). Such long latencies of locomotor changes suggest that they may be related to the visual rather than to the acoustic component of the distractions. 

Our analyses provided evidence for a link between distracter-induced gait changes and cognition. All our cognitive measures, including executive functions, visual-constructive skill, visual memory, visual planning, and subjects' verbal response errors, were poorer in seniors than in young subjects, and three of them—notably executive functions—explained partly or fully the effects of distracters on locomotion. In contrast, head-movements measures did not differ between young and older subjects except for the reaction time, and they did not contribute to the explanation of distracter effects. Since some but not all cognitive measures were associated with distracter effects, those effects cannot be attributed to generalized slowing in old age [33], but rather seem to reflect the decline of *specific* cognitive functions. Since our cognitive measures explained the distracter effects partly but not fully, those effects are probably linked to additional factors, including noncognitive factors like poor vision and reduced eye/head mobility [34, 35] or difficulties to reintegrate sensory inputs following a perturbation [36]. However, the observed age-related changes seem not linked to a decline of physical abilities due to sarkopenia, osteoporosis, and so on [37], since we found no evidence for such decline in the five unperturbed steps before the distraction. 

The induced change of the spatiotemporal gait pattern in the present study is reminiscent of the change observed in earlier work under dual-task conditions [2, 14, 38, 39] except that deficits of dual-task walking emerged both in young *and* in elderly subjects; they were larger in the elderly when the non-walking task had a high visual demand [12, 14, 15]. We have attributed the deficits of dual-task walking to limitations of executive functions [14]: visual processing for locomotion must be coordinated with that for another task, which is more difficult in old age because of prefrontal shrinkage [16]. The same interpretation could hold for the distracter-induced changes in the present work. Thus, both paradigms may call upon executive functions to coordinate two streams of visual information, which could be more challenging for older than for young subjects, and thus yield *larger* deficits in seniors when the second task is continuous (i.e., dual-task paradigm) and yield deficits only in seniors when it is brief (i.e., distracter paradigm).

Since lower-limb proprioception deteriorates in old age [40, 41], it has been argued that seniors rely increasingly on vision to maintain balance and that this limits their performance in dual-task walking [34, 42]. We reasoned that the same interpretation might also hold for distracter effects in the present study and introduced condition VIB to find out: If an age-related increase of distracter effects is related to poor proprioception, that increase should be even more pronounced when lower-limb proprioception is additionally degraded by vibration. However, our data provide little credibility for this view. Vibration did not influence locomotion in young subjects, and its influence on older subjects was quite limited: distracter effects manifested about 2 s earlier, but their magnitude remained unaltered. We attribute the faster effects in condition VIB to unspecific arousal rather than to an increased dependence on vision.

As pointed out above, the present distracter paradigm shares common features with everyday scenarios, and the observed effects on gait could therefore well have practical implications. When executive functions are challenged not only by old age, but additionally by diseases like stroke or dementia [43, 44], the detrimental effects on locomotion could well be amplified and thus precipitate accidental falls.

## Figures and Tables

**Table 1:** Subjects' gender anthropometric characteristics (means ± standard deviations).

	Older (*n* = 12)	Young (*n* = 12)
Males/females	6/6	5/7
Age (years)	68.17 ± 4.23	25.58 ± 2.75
Height (cm)	169.75 ± 7.24	174.50 ± 6.83
Weight (kg)	72.50 ± 9.55	69.75 ± 11.04
BMI (kg/m²)	25.08 ± 2.10	22.77 ± 2.33

**Table 2:** Outcome of linear regression analyses.

(a) Simple linear regression with regress age		*t*(22)	*P*
Step duration		−4.97	.0000
CV step duration		−2.24	.0356
Leg rotation		4.19	.0003
CV leg rotation		−3.11	.0051

(b) Multiple linear regression	Regressor^1^	*t*(22)	*P*

Step duration	Executive function	2.98	.0073
CV step duration	Executive function	8.09	.0000
	Age	2.69	.0149
	Psychomotor speed	3.37	.0034
Leg rotation	Age	3.05	.0063
	Psychomotor speed	−2.57	.0183
CV leg rotation	Executive function	2.22	.0384
	Visual memory	−2.26	.0357

^1^Only significant regressors are shown.
